# 544. Outpatient Remdesivir Use at a Stand Alone Children's Hospital

**DOI:** 10.1093/ofid/ofad500.613

**Published:** 2023-11-27

**Authors:** Brittany Rodriguez, Grant T Stimes, Tanya J Hilliard, Lisa Forbes Satter, Flor M Munoz

**Affiliations:** Texas Children's Hospital, Houston, Texas; Texas Children's Hospital, Houston, Texas; Texas Children's Hospital, Houston, Texas; Baylor College of Medicine, Houston, Texas; Baylor College of Medicine, Houston, Texas

## Abstract

**Background:**

When remdesivir was approved for non-hospitalized, high-risk patients with mild to moderate COVID-19, Texas Children’s Hospital (TCH) implemented an outpatient clinic for eligible patients to receive remdesivir infusions. There are limited data evaluating the use of outpatient remdesivir in pediatric patients. We describe the clinical characteristics and outcomes of the patients treated with outpatient remdesivir at TCH.

**Methods:**

Patients that were referred to receive outpatient remdesivir from June 7, 2022 to April 5, 2023 were included. Information collected includes demographics, comorbidities, refusal reason, adverse events, emergency center (EC) visit or admission within 14 days after final dose, and EC visit or admission within 14 days of referral if patients did not receive remdesivir. Chi-square was used to determine differences between the groups.

**Results:**

There were 187 patients referred during the study period. The median age of all referred patients was 8.8 years (IQR 4.84-12.96 years). The median time from reported symptom onset to dose 1 of remdesivir was 2 days (IQR 1-4 days). The comorbidities of all patients are listed in Figure 1. Out of the 182 patients, 69 patients (37.9%) received remdesivir, 10 patients (5.5%) were admitted prior to receiving their first dose, and 107 (58.8%) were not treated. The most common refusal reason was improvement between the referral and treatment evaluation visit (Figure 2). Among those treated, 8 patients (11.6%) visited the EC resulting in 7 admissions (10.1%) within 14 days of their last dose of remdesivir, while 7 patients (6.5%) that were referred but did not receive remdesivir visited the EC resulting in 3 admissions (2.8%) within 14 days of their referral being placed (p=0.04). More serious underlying conditions (e.g. immunosuppression, chronic lung disease, etc.) were more common among treated patients vs. untreated patients (p=0.00002).

**Figure:**
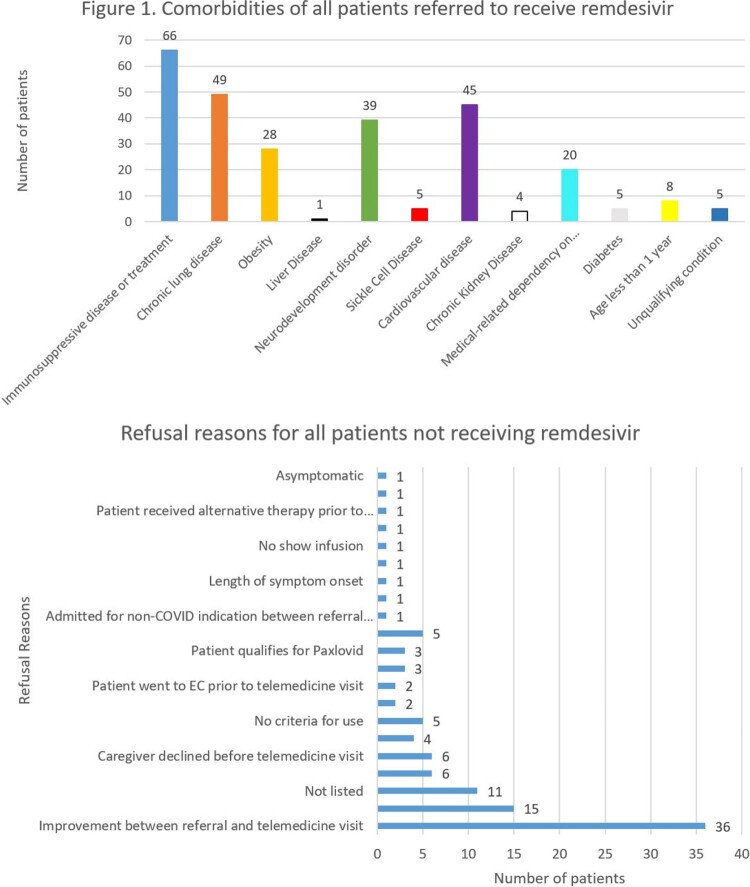

**Conclusion:**

Outpatient remdesivir was well tolerated among pediatric patients. In our series, the majority of high- risk patients receiving outpatient remdesivir treatment did not require subsequent EC visit or hospitalization. More robust data are needed to determine the clinical efficacy of this treatment for pediatric patients in the outpatient setting.

**Disclosures:**

**Lisa Forbes Satter, MD**, ADMA: Advisor/Consultant|CsL Behring: Advisor/Consultant|Grifols: Advisor/Consultant|incyte: Advisor/Consultant|Pharming: Advisor/Consultant|Takeda: Advisor/Consultant **Flor M. Munoz, MD, MSc**, CDC respiratory virus surveillance: Grant/Research Support|Gilead: Grant/Research Support|Moderna, sanofi, aztra zeneca, Merck, GSK: Advisor/Consultant|NIH: DSMB|NIH COVID-19 vaccines in pregnancy: Grant/Research Support|Pfizer Pediatric COVID-19 vaccines: Grant/Research Support|Pfizer, Dynavax, Monderna, Meissa, NIH: DSMB

